# Risk Factors Influencing Right and Left Ventricular Variables Assessed with Gated Cadmium–Zinc–Telluride Equilibrium Radionuclide Angiocardiography in Oncology Patients

**DOI:** 10.3390/diagnostics15101274

**Published:** 2025-05-17

**Authors:** Olav Monsson, Marc Nielsen, Thomas Kümler, Christian Haarmark, Bo Zerahn

**Affiliations:** 1 Department of Nuclear Medicine, Herlev og Gentofte Hospital, 2730 Herlev, Denmark; marc.oestergaard.nielsen@regionh.dk (M.N.); christian.eickhoff.haarmark.nielsen.01@regionh.dk (C.H.); bo.zerahn@regionh.dk (B.Z.); 2 Department of Radiology, Odense University Hospital, 5000 Odense, Denmark; 3 Research Unit of Radiology, Department of Clinical Research, University of Southern Denmark, 5000 Odense, Denmark; 4 Complications Research, Steno Diabetes Center Copenhagen, 2730 Herlev, Denmark; thomas.kuemler@regionh.dk; 5 Department of Cardiology, Herlev-Gentofte Hospital, 2730 Herlev, Denmark; 6 Department of Clinical Medicine, Copenhagen University, 2200 Copenhagen, Denmark

**Keywords:** CZT SPECT, equilibrium radionuclide angiography, cardiac function, left ventricular ejection fraction, right ventricular ejection fraction

## Abstract

**Background**: Left ventricular ejection fraction remains the primary focus in cardiac monitoring for oncology patients undergoing potentially cardiotoxic chemotherapy, while right ventricular function is seldom examined. This study evaluates how established risk factors for left ventricular dysfunction affect right ventricular function. **Methods**: This retrospective cohort study included 1770 patients undergoing cadmium–zinc–telluride equilibrium radionuclide angiocardiography before chemotherapy. Patients were categorized based on risk factors for left ventricular dysfunction—diabetes (DM), atrial fibrillation (AF), coronary heart disease (CHD), and previous oncological therapy—and compared to controls using independent *t*-tests. **Results**: Patients with previous oncological therapy exhibited a significantly lower right ventricular end-diastolic volume (RVEDV) (mean difference: −4.4 mL/m^2^, 95% CI: −6.1 to −2.7, *p* < 0.001), lower right ventricular end-systolic volume (RVESV) (−2.3 mL/m^2^, 95% CI: −3.4 to −1.2, *p* < 0.001), and lower right ventricular stroke volume (RVSV) (−2.1 mL/m^2^, 95% CI: −3 to −1.2, *p* < 0.001). In patients with CHD, there was a higher right ventricular ejection fraction (RVEF) (3.0 mL/m^2^, 95% CI: 0.8 to 5.2, *p* < 0.01), whereas patients with DM had lower RVEDV (−5.1 mL/m^2^, 95% CI: −9.2 to −1, *p* < 0.05) and RVESV (−3.0 mL/m^2^, 95% CI: −5.5 to −0.4, *p* < 0.05). No ventricular variables differed from the control group among patients with AF. **Conclusions**: Risk factors known to affect the left ventricle also impacted the right ventricle, with the exception of AF.

## 1. Introduction

Cardiotoxicity is a significant concern in the administration of antineoplastic drugs and underscores the complexity of balancing effective cancer treatment against the potential for adverse cardiac events [1,2,3]. According to guidelines [2], routine assessment is primarily centered on left ventricular function assessed by left ventricular ejection fraction (LVEF) along with other left ventricular variables such as diastolic function and strain, whereas the right ventricular function is somewhat overlooked in part due to the lack of available, practical, and standardized assessment methods, but also to an extent limited recognition of its clinical importance in many pathological conditions [4]. Nonetheless, growing evidence highlights the right ventricle’s importance in cardiac efficiency and hemodynamic stability, leading to increased focus on its function [5,6].

The most widely used technology to assess cardiac function is two-dimensional transthoracic echocardiography (2D-TTE) recommended in guidelines [2]. Due to its anatomy, assessment of the right ventricle using 2D-TTE has proven difficult [4]. Three-dimensional transthoracic echocardiography (3D-TTE) offers enhanced accuracy and detail along with diastolic function and strain. Despite its advancements, 3D-TTE remains challenging for accurate volumetric assessment of the right ventricle [7].

Emerging insights into cardiac physiology suggest that the right ventricle plays an equally vital role in the pathophysiology of various cardiac and systemic conditions, including coronary heart disease (CHD), diabetes (DM), atrial fibrillation (AF), and the aftermath of potential cardiotoxic oncological interventions [8,9,10,11,12]. This necessitates a broader understanding of cardiac health that encompasses both ventricles. Moreover, the advent of advanced imaging techniques, such as gated cadmium–zinc–telluride single-photon emission computed tomography equilibrium radionuclide angiocardiography (CZT-SPECT-ERNA), has provided new avenues for detailed cardiac assessment, enabling a more nuanced evaluation of ventricular function beyond conventional measures.

Equilibrium radionuclide angiography (ERNA) is a widely recognized and non-invasive imaging [13]. Notably, CZT-SPECT-ERNA stands out due to its practicality, operator independence, and high reproducibility [14,15,16]. Recent research has further optimized this technique by reducing the radiotracer dose while maintaining diagnostic value [17].

We hypothesize that while the right and left ventricles are interconnected but the extent to which common risk factors such as CHD, DM, AF, and previous oncological therapy affect right ventricular function remains insufficiently characterized. This study aims to quantify these effects and determine whether they differ from established left ventricular changes. By performing so, it seeks to elucidate the broader implications of these risk factors on cardiac function, with a particular emphasis on the right ventricle.

As a secondary objective, this study aims to assess whether the presence of these left ventricular risk factors significantly alters left ventricular function. By quantifying these differences, we seek to justify the exclusion of patients with these comorbidities in previously published normative data [18,19].

## 2. Materials and Methods

### 2.1. Study Design, Population, and Data Collection

This is a retrospective cohort study of patients referred from a tertiary oncological department at Herlev-Gentofte University Hospital to undergo gated CZT-SPECT-ERNA for baseline evaluation before initiating antineoplastic treatments with potential cardiotoxicity. The patient population is described in the publications of Haarmark et al. and Hansen et al. [18,19], and was originally collected between October 2012 and 30 September 2014.

Patients with CHD, DM, AF, and previous oncological therapy comprised four different test groups, while the control group had none of these conditions. Patients were excluded if they had pleural effusions, prior lung resection, or more than one of the risk factors (CHD, DM, AF or previous oncological treatment) in order to ensure more homogeneous comparison groups.

Screening was conducted by manual reading of the patient’s electronic hospital records (EPJ) and the Shared Medication Record (FMK). CHD was defined as a history of myocardial infarction, coronary artery bypass grafting, or coronary revascularization. DM was defined as current or previous elevation of hemoglobin A1c > 48 mmol/mol (HbA1c) or receiving antidiabetic treatment. AF was defined as having a medical history of prior or current atrial fibrillation. Previous oncological therapy was defined as any history of treatment with any chemotherapeutic agents and/or radiation therapy to the thoracic region.

Left and right ventricular ejection fractions (LVEF, RVEF), end-systolic volumes (LVESV, RVESV), end-diastolic volumes (LVEDV, RVEDV), and stroke volumes (LVSV, RVSV) were assessed using gated CZT-SPECT-ERNA with 99mTc-labeled human serum albumin (HSA) as the radiotracer.

**Figure 1:**
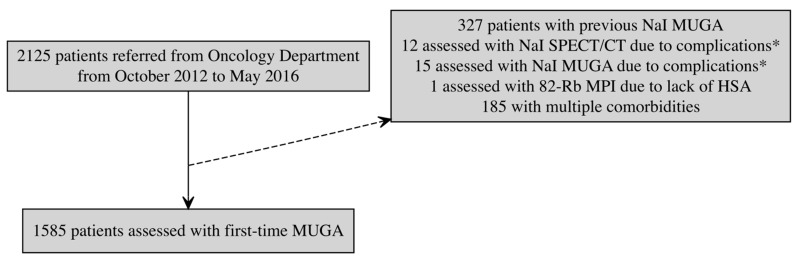
Flow chart of the study population. Solid arrow indicate inclusion in the main study cohort, while dashed arrow indicate exclusion. * Complications include inability to abduct the left shoulder (*n* = 12), a too large thoracic circumference (*n* = 6), gating and trigger issues (*n* = 2), claustrophobia (*n* = 2), camera malfunction (*n* = 2), sarcoma proximally to the heart (*n* = 1), heart malrotation (*n* = 1), and lack of staff (*n* = 1).

To enhance statistical power, the inclusion period was extended to May 2016. Hence, patients were included if the first CZT-SPECT-ERNA scan was performed between October 2012 and May 2016, and excluded if they were assessed with alternative methods (see Figure 1).

### 2.2. Ethical Considerations

The study was approved by the Danish data protection Agency (R-24081714).

The corresponding author had full access to all the data in this study and takes responsibility for its integrity and the accuracy of the analysis.

### 2.3. Gated CZT-SPECT-ERNA Procedure

**Figure 2:**
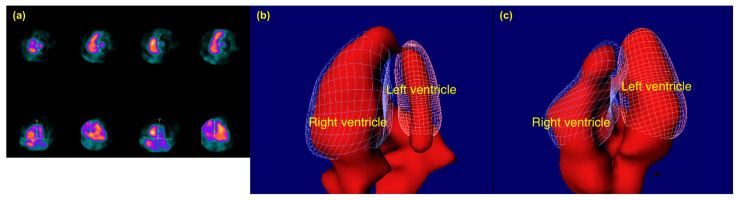
Representative end-diastolic CZT-SPECT-ERNA frames showing (**a**) two-dimensional adjustment mode, (**b**) dilated right ventricle, and (**c**) normal right ventricle as seen an apical four-chamber-like view. Images are representative of the assessment for RV dimensions in this study. In panel (**a**), colors are based on standardized uptake values confined by the color template (thermal), dotted lines indicate the region of interest, while solid lines with circles or squares denote anatomical landmarks such as the interventricular septum, tricuspid valve, and mitral valve.

Equilibrium radionuclide angiocardiography (ERNA) was performed using a cadmium zinc telluride (CZT) gamma camera system, GE Discovery 530c (GE Healthcare, Milwaukee, WI, USA). A dose of 550 MBq 99mTc-labeled HSA was administered intravenously. Patients were positioned supine with both arms extended above their heads. Gated data acquisition was obtained with 16 image frames (bins) per cardiac cycle, requesting 600 accepted beats, and a 20% energy window centered on 140 keV. Subsequently, the acquired data were processed and analyzed using Xeleris 3 Imaging workstation reorientation software (GE Healthcare, Milwaukee, WI, USA, version no. 3.0562) and Cedars-Sinai QBS processing software (Cedars-Sinai, Los Angeles, CA, USA, revision 2009.0) to generate spline-based time-activity curves for the ventricles, which were then used to calculate end-diastolic and -systolic volumes for both right and left ventricle. Figure 2 illustrates representative end-diastolic frames from patients with normal and dilated RVs.

Each acquisition was analyzed twice by two experienced technologists. If LVEF differed more than two percentage points between the two analyses, repeated processing was performed until sufficient agreement was obtained. In cases where RVEF differed by more than ten percentage points between two replicate analyses, a third analysis was performed, and the two most concordant values were used for reporting.

### 2.4. General Considerations

Right ventricular volumes are dependent on body surface [20] and were therefore corrected for this using Mostellar corrections.

### 2.5. Statistical Analysis

For continuous variables, independent *t*-tests were employed to assess statistical differences between the groups. For dichotomized variables, Chi-square tests were used.

Ad hoc univariate linear regression analyses were performed to identify candidate predictors of right ventricular volumes. Variables with *p* < 0.25 were considered for inclusion in multivariate models. In addition, CHD, AF, diabetes, and previous oncological therapy were included in all models regardless of univariate significance, due to their clinical relevance. Model performance was compared using adjusted R-squared values and ANOVA.

Adjusted β coefficients and 95% confidence intervals were visualized using forest plots. To further illustrate the independent effect of selected predictors (diabetes and previous oncological therapy), we generated adjusted effect plots based on model-derived predicted values, using the ggeffects package in R.

Extreme outliers were identified and excluded using the 3 × IQR rule.

All analyses were performed using RStudio 2022.07.1 + 554 “Spotted Wakerobin” Release and R version 4.3.1, and a *p*-value < 0.05 was considered statistically significant.

## 3. Results

The patient flow chart is presented in Figure 1. The study population detailed in Table 1, consists of 1770 patients of whom 811 were categorized as the control group and the remainder as having one of the risk factors.

**Figure 3:**
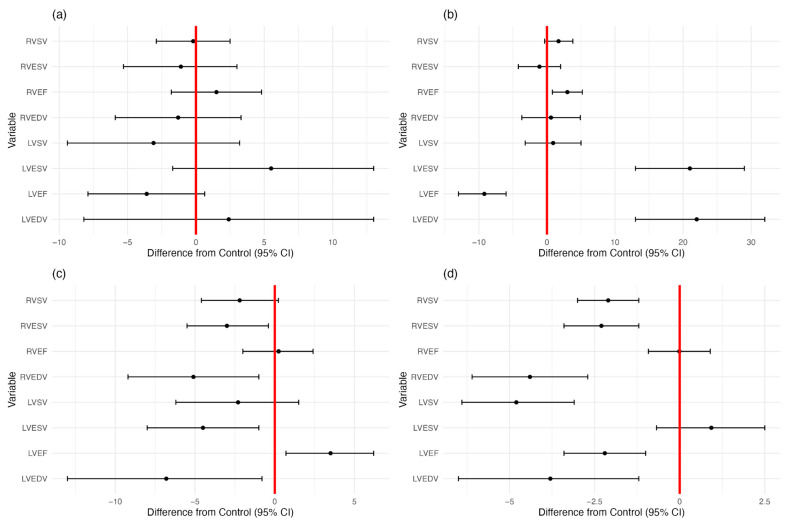
Dot plot with error bars of patients with (**a**) AF, (**b**) CHD, (**c**) DM, and (**d**) previous oncological therapy and their influence on left and right ventricular variables.

Comparisons of the control group with AF, CHD, diabetes, and previous oncological therapy are presented in Table 2 and Figure 3.

Patients with previous oncological therapy had a lower LVEDV (*p* = 0.0051), leading to a lower LVEF (*p* < 0.001) and LVSV (*p* < 0.001). The same can be seen for RVEDV (*p* < 0.001). However, unlike the left ventricle, RVESV was also reduced (*p* < 0.001), leading to an unchanged RVEF.

For patients with DM, a statistically significant lower LVEDV (*p* = 0.027) and LVESV (*p* = 0.012) was noted; however, the lower LVESV was the most pronounced, leading to a higher LVEF (*p* = 0.015). Similar differences were seen in the right ventricle, except for an unchanged RVEF. RVEDV was lower in DM patients (*p* = 0.015) along with RVESV (*p* = 0.024).

For patients with CHD, the RVEF was significantly higher (*p* = 0.0082), despite RVESV and RVEDV remaining statistically unchanged. Differences can be seen in the left ventricle through a lower LVEF (*p* < 0.001), attributed to a higher value of LVESV (*p* < 0.001) compared to LEDV (*p* < 0.001).

In the AF group, no significant differences from the control group were observed for either left or right ventricular values.

To assess the contribution of CHD, AF, DM, and previous oncological therapy, we compared a core regression model including age, blood pressure, heart rate, gender, hypertension, hypercholesterolemia, and smoking with a full model that also included the above clinical variables. For Right EDV, the addition of CHD, AF, diabetes, and previous oncological therapy led to a statistically significant improvement in model fit (adjusted R^2^ = 0.198 vs. 0.194, *p* = 0.013). For Right ESV, the full model showed only a minimal and statistically non-significant increase in explanatory power (adjusted R^2^ = 0.207 vs. 0.206, *p* = 0.233) (see Supplementary Table S1).

**Figure 4:**
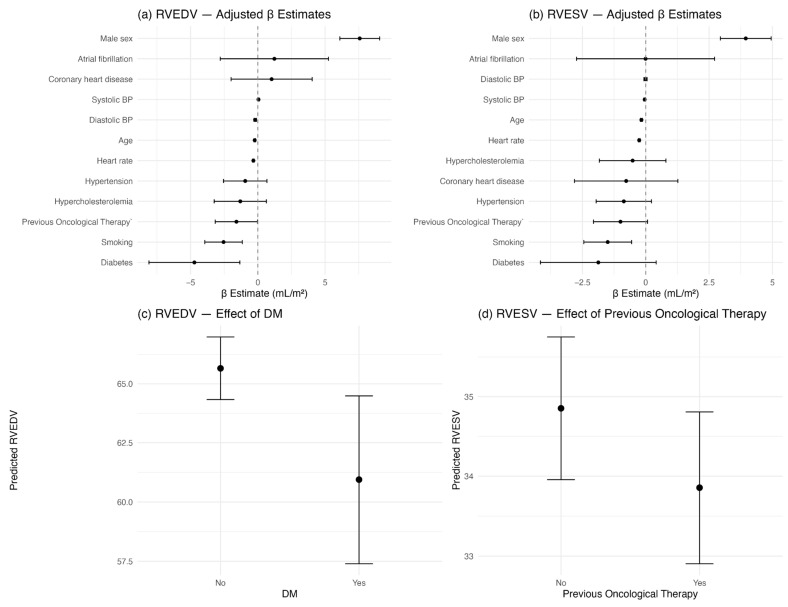
Association between clinical predictors and right ventricular volumes. (**a**) and (**b**) Forest plots showing adjusted β coefficients (with 95% confidence intervals) from multivariate linear regression models for RVEDV and RVESV, respectively. (**c**) Model-predicted RVEDV for patients with and without diabetes, adjusted for all other covariates. (**d**) Model-predicted RVESV for patients with and without previous oncological therapy, adjusted for all other covariates. All values are indexed to body surface area (mL/m^2^).

After adjusting for age, systolic blood pressure, diastolic blood pressure, heart rate, gender, and other cardiovascular risk factors, patients with diabetes had a predicted RVEDV of 60.9 mL/m^2^ (95% CI: 57.4 to 64.5), compared to 65.7 mL/m^2^ (95% CI: 64.3 to 67.0) in patients without diabetes. Similarly, patients with previous oncological therapy had a predicted RVESV of 33.9 mL/m^2^ (95% CI: 32.9 to 34.8), compared to 34.9 mL/m^2^ (95% CI: 34.0 to 35.8) in those without previous oncological therapy (Figure 4).

Results from the corresponding univariate analyses are presented in Supplementary Table S2.

## 4. Discussion

This study evaluates how different comorbidities affecting the left ventricle—CHD, AF, DM, and previous oncological therapy—influence the same cardiac variables for the right ventricle, utilizing gated CZT-SPECT-ERNA.

Patients with previous oncological therapy exhibited lower RVEDV, RVESV, and RVSV. A systematic review by Kariyanna et al. [21] reported a reduced RVEF in the follow-up of breast cancer patients receiving anthracycline and/or trastuzumab. A study by Grover et al. [22] using MRI in the follow-up of anthracycline and/or trastuzumab treatment, reported a reduction in RVEF, though an increase in right ventricular ESV and EDV, indexed by body surface area, at 12 month follow-up. The discrepancy from this study could be the result of differences in imaging techniques or lack of discrimination between the different oncological treatments in regard to potential cardiotoxic effects. Few studies have examined the volumetric impact on the right ventricle, although reductions in other right ventricular function parameters have been reported [23,24].

For patients with previous oncological therapy, our study found a lower left EDV and a related lower SV and LVEF. This agrees with the current literature [25,26]. The aforementioned MRI study by Grover et al. [22], showed a higher left ventricular EDV and ESV, and a lower LVEF.

The regression analysis demonstrated that gender, age, and heart rate were the most consistent contributors to variability in right ventricular volumes, in line with physiological expectations [27,28]. In addition, smoking was negatively associated with both EDV and ESV. Importantly, diabetes was independently associated with a significantly lower right ventricular EDV, and previous oncological therapy showed a modest but statistically significant association with lower EDV and a borderline effect on ESV. These findings from the multivariable analysis reinforce the primary hypothesis of the study—that specific clinical risk factors traditionally associated with left ventricular dysfunction may also affect right ventricular remodeling, albeit in different ways.

Noteworthy for this study is its cross-sectional approach, as opposed to the aforementioned follow-up studies. This also might be relevant for the other risk factors, as this study evaluates patients in a relatively stable disease state.

For patients with CHD, the observed lower LVEF due to a higher LVESV and LVEDV is consistent with the existing literature [29,30]. Ischemia-induced myocyte loss and fibrosis likely contribute to impaired contractility, increased ventricular stiffness, and dilatation, culminating in reduced LVEF.

However, the right ventricle does not display the same features. The right ventricle exhibits a higher EF, though no statistically significant differences in ESV or EDV. This might reflect the right ventricle’s compensatory capacity and resilience towards ischemia [31,32]. A study by van Wolferen et al. [33] of patients with idiopathic pulmonary arterial hypertension found that right ventricular dilatation and impaired left ventricular filling were associated with worse outcomes over a one-year period, although this population differs from ours. Considering the primary factor for pulmonary hypertension being left ventricular failure, the finding by van Wolferen et al. could further support our findings. This finding may indicate the right ventricle’s adaptive response to ischemia, potentially compensating for left ventricular dysfunction via increased contractility or improved coupling with pulmonary circulation.

In diabetic patients, lower RVEDV and RVESV, with preserved RVEF, align with findings from MRI studies [34]. These findings suggest a parallel diastolic dysfunction in the right ventricle, highlighting the systemic nature of diabetes-related cardiomyopathy. In patients with cardiomyopathy, gated CZT-SPECT-ERNA has been shown to be accurate and reproducible when measuring right ventricular function and is considered a good alternative to gold-standard cardiac assessment [35].

A lower left ventricular EDV and ESV, accompanied by a higher LVEF in diabetic patients in our study, are consistent with early diastolic dysfunction features seen in diabetic cardiomyopathy [36]. Diabetes is well-documented to affect myocardial compliance, with microvascular damage, interstitial fibrosis, and glycation of myocardial proteins contributing to stiffened ventricles and impaired filling [37,38].

While prior studies have demonstrated lower LVEF and higher LVESV in AF patients [39,40], this study’s findings suggest these differences may not be universally detectable, potentially influenced by differences in AF chronicity, ventricular remodeling, or underlying pathophysiology.

For patients with AF, the lack of significant differences in ventricular function compared to controls might reflect limitations in the sensitivity of CZT-SPECT-ERNA imaging in this subgroup. It is well known that AF is associated with dynamic changes in stroke volume and ventricular filling due to irregular ventricular response and loss of atrial contribution to cardiac output [41]. However, the variability in ventricular filling across cardiac cycles in AF may complicate accurate assessment, particularly in methods dependent on consistent temporal data acquisition like CZT-SPECT-ERNA. The wide confidence intervals observed further underscore the variability and potential measurement uncertainty in this population.

In addition to the primary risk factors examined, smoking was independently associated with reduced RVEDV and RVESV in our multivariate analysis. Although some studies in patients with advanced pulmonary disease or long-term smoking exposure have reported right ventricular dilation and dysfunction [10], our findings may reflect an earlier or subclinical pattern of RV remodeling. This could involve fibrosis, microvascular injury, or impaired myocardial compliance, leading to lower filling volumes without affecting ejection fraction. Supporting this interpretation, a study by Chahal et al. using cardiac MRI in the MESA cohort found that former smokers with greater emphysema had smaller RV end-diastolic volumes after adjusting for left ventricular volume, suggesting that smoking-related myocardial remodeling may present as reduced RV volumes rather than dilation in certain contexts [42].

### Limitations

Several limitations of this study warrant consideration. First, being a retrospective, single-center study narrows down the generalizability to other contexts. Second, we chose not to correct for the large number of variables analyzed, due to the nature of the explorative study. As such, there is an evident risk for type I errors. Third, since this study also focuses on right ventricular values, it would have been interesting also to evaluate chronic obstructive pulmonary disease (COPD), pulmonary embolism (PE), and pulmonary hypertension (PH) as potential risk factors for reduced cardiac function. Fourth, potential confounding factors, such as medication use, disease duration, and comorbidities such as valvular disease, were not fully accounted for, which may influence the observed differences. As an example, there is no differentiation between anthracyclines and other types of chemotherapy. Further, there are differences in baseline characteristics (age, height, and weight) which obscures the interpretation of the ventricular volumes. Additionally, laboratory data on renal function (e.g., eGFR) were not available, although renal impairment is a recognized cardiovascular risk factor and potential confounder in volumetric cardiac assessments.

Although this study aims to isolate the effects of CHD, DM, AF, and oncological treatment on cardiac function, patients often have other comorbidities that may independently or synergistically influence outcomes. Additionally, variability in malignancies and metastatic disease introduces potential selection bias.

## 5. Conclusions

This study found that risk factors influencing the left ventricle could also influence the right ventricle. Notably, previous oncological therapy and DM appear to impact the right ventricle to a similar extent as the left.

Future studies should examine specific risk factors for right ventricular dysfunction to further investigate volumetric changes and their clinical significance, including potential applications for treatment monitoring and outcome prediction in cancer therapy and the impact of medical treatment and multimorbidity on right ventricular function.

## Figures and Tables

**Table 1:** Patient characteristics.

Variable	Control (*n* = 811)	AF (*n* = 54)	CHD (*n* = 104)	DM (*n* = 81)	Previous Oncological Therapy (*n* = 535)
Gender (men)	367 (45%)	30 (56%)	66 (63%)	58 (72%)	103 (19%)
Age	61 (±15)	73 (±7) *	69 (±11) *	67 (±8) *	62 (±11) *
Height (women) [cm]	166 (±7)	167 (±5)	164 (±6)	164 (±8)	166 (±6)
Height (men) [cm]	178 (±7)	179 (±5)	176 (±6) *	177 (±7)	180 (±7)
Weight (women) [kg]	70 (±14)	72 (±12)	68 (±12)	77 (±25)	70 (±14)
Weight (men) [kg]	82 (±14)	81 (±9)	80 (±12)	87 (±15) *	83 (±15)
Systolic BP [mmHg]	129 (±19)	128 (±20)	129 (±20)	127 (±16)	125 (±18) *
Diastolic BP [mmHg]	77 (±10)	76 (±11)	75 (±10)	73 (±10) *	76 (±10)
Heart rate [bpm]	75 (±13)	76 (±17)	73 (±15)	77 (±15)	77 (±14) *
Arterial hypertension	229 (28%)	32 (59%)	64 (62%)	55 (68%)	138 (26%)
Hypercholesterolemia	95 (12%)	21 (39%)	56 (54%)	38 (47%)	56 (10%)
Smoking (prior or current)	350 (43%)	27 (50%)	65 (63%)	47 (58%)	208 (39%)

* *p*-value < 0.05.

**Table 2:** T-test comparison for each risk factor.

Variable	Left Ventricle	Right Ventricle
AF		
EDV	2.4 (−8.2–13, *p* = 0.65)	−1.3 (−5.9–3.3, *p* = 0.56)
ESV	5.5 (−1.7–13, *p* = 0.13)	−1.1 (−5.3–3, *p* = 0.58)
EF	−3.6 (−7.9–0.64, *p* = 0.094)	1.5 (−1.8–4.8, *p* = 0.37)
SV	−3.1 (−9.4–3.2, *p* = 0.33)	−0.2 (−2.9–2.5, *p* = 0.88)
CHD		
EDV	22 (13–32, *p* < 0.001)	0.6 (−3.7–4.9, *p* = 0.78)
ESV	21 (13–29, *p* < 0.001)	−1.1 (−4.2–2, *p* = 0.48)
EF	−9.2 (−13–(−6), *p* < 0.001)	3 (0.79–5.2, *p* = 0.008)
SV	0.9 (−3.2–5, *p* = 0.66)	1.7 (−0.35–3.8, *p* = 0.1)
DM		
EDV	−6.8 (−13–(−0.8), *p* = 0.027)	−5.1 (−9.2–(−1), *p* = 0.015)
ESV	−4.5 (−8–(−1), *p* = 0.012)	−3 (−5.5–(−0.4), *p* = 0.024)
EF	3.5 (0.7–6.2, *p* = 0.015)	0.2 (−2–2.4, *p* = 0.83)
SV	−2.3 (−6.2–1.5, *p* = 0.23)	−2.2 (−4.6–0.23, *p* = 0.076)
Previous oncological therapy		
EDV	−3.8 (−6.5–(−1.2), *p* = 0.005)	−4.4 (−6.1–(−2.7), *p* < 0.001)
ESV	0.9 (−0.68–2.5, *p* = 0.26)	−2.3 (−3.4–(−1.2), *p* < 0.001)
EF	−2.2 (−3.4–(−1), *p* < 0.001)	−0.01 (−0.92–0.90, *p* = 0.98)
SV	−4.8 (−6.4–(−3.1), *p* < 0.001)	−2.1 (−3–(−1.2), *p* < 0.001)

## Data Availability

The data presented in this study are not publicly available due to Danish data protection regulations. Individual-level patient data were accessed through approval from Team for Journaldata, Region Hovedstaden (Project ID: R-24081714), under Section 46(2) of the Danish Health Act. Data are considered sensitive health information and cannot be shared publicly or anonymized for open release. Researchers who meet the criteria for access to confidential data can contact Team for Journaldata at journaldata.center-forsundhed@regionh.dk to request data access under similar regulatory approval.

## Supplementary Materials

The following supporting information can be downloaded at: https://www.mdpi.com/article/10.3390/diagnostics15101274/s1, Supplementary Table S1: Multivariate linear regression analysis; Supplementary Table S2: Univariate linear regression analysis.

## Abbreviations

The following abbreviations are used in this manuscript:

2D-TTE	Two-Dimensional Transthoracic Echocardiography
3D-TTE	Three-Dimensional Transthoracic Echocardiography
AF	Atrial Fibrillation
CHD	Coronary Heart Disease
COPD	Chronic Obstructive Pulmonary Disease
CZT	Cadmium–Zinc–Telluride
CZT-SPECT-ERNA	Cadmium–Zinc–Telluride Single-Photon Emission Computed Tomography Equilibrium Radionuclide Angiocardiography
DM	Diabetes Mellitus
EPJ	Elektronisk Patientjournal (Electronic Patient Record)
ERNA	Equilibrium Radionuclide Angiocardiography
FMK	Fælles Medicinkort (Shared Medication Record)
HbA1c	Hemoglobin A1c
HSA	Human Serum Albumin
LVEDV	Left Ventricular End-Diastolic Volume
LVEF	Left Ventricular Ejection Fraction
LVESV	Left Ventricular End-Systolic Volume
LVSV	Left Ventricular Stroke Volume
PE	Pulmonary Embolism
PH	Pulmonary Hypertension
RIS/PACS	Radiology Information System/Picture Archiving and Communication System
RVEDV	Right Ventricular End-Diastolic Volume
RVEF	Right Ventricular Ejection Fraction
RVESV	Right Ventricular End-Systolic Volume
RVSV	Right Ventricular Stroke Volume
SV	Stroke Volume
