# Supplementary material for: Risk Factors Influencing Right and Left Ventricular Variables Assessed with Gated Cadmium–Zinc–Telluride Equilibrium Radionuclide Angiocardiography in Oncology Patients
Source: Diagnostics (Basel). 2025 May 17;15(10):1274. doi: 10.3390/diagnostics15101274 (PMC12110102; doi:10.3390/diagnostics15101274)
Supplement: Supplementary file 1 [file diagnostics-15-01274-s001.zip › diagnostics-3609096-supplementary.pdf]

Supplementary Table S1. Multivariate linear regression analysis

**Predictor**	**β (95% CI)**	**p-value**
Right EDV		
Age	-0.23 (-0.29, -0.17)	< 0.001
Systolic blood pressure	0.05 (-0.00, 0.10)	0.070
Diastolic blood pressure	-0.20 (-0.29, -0.11)	< 0.001
Heart Rate	-0.33 (-0.38, -0.28)	< 0.001
Gender (Male)	7.57 (6.09, 9.05)	< 0.001
Hypertension	-0.94 (-2.55, 0.68)	0.26
Hypercholesterolemia	-1.30 (-3.24, 0.64)	0.19
Smoking (prior or current)	-2.55 (-3.94, -1.16)	< 0.001
AF	1.23 (-2.79, 5.25)	0.55
CHD	1.03 (-1.99, 4.04)	0.50
DM	-4.72 (-8.09, -1.34)	0.006
Previous oncological therapy	-1.59 (-3.17, -0.02)	0.047
Right ESV		
Age	-0.17 (-0.21, -0.13)	< 0.001
Systolic blood pressure	-0.04 (-0.08, -0.01)	0.020
Diastolic blood pressure	-0.01 (-0.07, 0.05)	0.77
Heart Rate	-0.26 (-0.29, -0.22)	< 0.001
Gender (Male)	3.95 (2.95, 4.95)	< 0.001
Hypertension	-0.87 (-1.96, 0.23)	0.12
Hypercholesterolemia	-0.52 (-1.83, 0.80)	0.44
Smoking (prior or current)	-1.50 (-2.44, -0.56)	0.0020
AF	-0.01 (-2.73, 2.72)	0.99
CHD	-0.77 (-2.81, 1.27)	0.46
DM	-1.87 (-4.16, 0.41)	0.11
Previous oncological therapy	-1.00 (-2.06, 0.07)	0.067

Core model includes age, blood pressures, heart rate, gender, hypertension, hypercholesterolemia, and smoking. Full model additionally includes CHD, AF, diabetes, and prior chemotherapy.

Supplementary Table S2. Univariate linear regression analysis

Predictor	β (95% CI)	p-value
Right EDV		
Age	-0.21 (-0.27, -0.16)	< 0.001
Systolic blood pressure	-0.07 (-0.11, -0.03)	< 0.001
Diastolic blood pressure	-0.22 (-0.29, -0.14)	< 0.001
Heart Rate	-0.38 (-0.43, -0.33)	< 0.001
Gender (Male)	6.55 (5.06, 8.05)	< 0.001
Hypertension	-2.50 (-4.06, -0.94)	0.002
Hypercholesterolemia	-2.21 (-4.11, -0.30)	0.023
Smoking (prior or current)	-1.97 (-3.47, -0.46)	0.010
Right ESV		
Age	-0.19 (-0.23, -0.15)	< 0.001
Systolic blood pressure	-0.08 (-0.11, -0.05)	< 0.001
Diastolic blood pressure	-0.12 (-0.17, -0.07)	< 0.001
Heart Rate	-0.27 (-0.30, -0.23)	< 0.001
Gender (Male)	3.29 (2.26, 4.32)	< 0.001
Hypertension	-2.55 (-3.61, -1.49)	< 0.001
Hypercholesterolemia	-1.98 (-3.27, -0.68)	0.003
Smoking (prior or current)	-1.39 (-2.41, -0.36)	0.008
